# Combining species distribution modeling and field surveys to reappraise the geographic distribution and conservation status of the threatened thin-spined porcupine (*Chaetomys subspinosus*)

**DOI:** 10.1371/journal.pone.0207914

**Published:** 2018-11-27

**Authors:** Gastón Andrés Fernandez Giné, Deborah Faria

**Affiliations:** Departamento de Ciências Biológicas, Laboratório de Ecologia e Conservação de Espécies Ameaçadas, Universidade Estadual de Santa Cruz, Ilhéus, Bahia, Brazil; Fred Hutchinson Cancer Research Center, UNITED STATES

## Abstract

The threatened thin-spined porcupine (*Chaetomys subspinosus*), a forest-specialist endemic to the Brazilian Atlantic forest, was rarely detected in the wild during the 20^th^ century. Previous geographic distribution assessments were carried out nearly three decades ago and were based on interview data. We performed extensive field surveys (based on active search and interviews), a literature review, and species distribution modeling to predict and validate a more reliable picture of its geographic distribution and environmental suitability gradient. We identified the main predictors of species’ incidence, its conservation status, and pinpointed key areas for species conservation. Our results indicated that *C*. *subspinosus* is distributed continuously in the Atlantic forest from southeastern Espirito Santo to central-eastern Sergipe state, totaling 104,326 km^2^ of occurrence area, although only 3,299 km^2^ (13.3%) is currently represented by native forests (species habitat). *C*. *subspinosus* was absent or at least so rare that it was not detected in more than half of the locations sampled by interviews (53.5%). Our results suggest that populations are sensitive to climatic conditions and habitat loss, becoming abruptly rarer when the remaining forest cover reaches less than 10% area within a region (~ 5,000 km^2^ scale). This result indicates that the high deforestation level of the Atlantic forest is already close to the limit of regional species resistance. Bahia state still harbors the bulk of the remaining forest with high climatic suitability, and generally under low levels of legal protection. Herein we highlight priority areas and research gaps that could guide decision makers to promote conservation strategies for this threatened species.

## Introduction

Species are disappearing at unprecedented rates, and a reliable picture of species distribution is needed to assess their conservation status and planning conservation efforts. Unfortunately, decision-makers often lack such crucial information, particularly for tropical species that together comprise the bulk of species threatened by extinction [1,2]. Although ecological niche modeling (ENM) has been increasingly applied as a tool to assess and predict species distribution [3–5], conservation status [6–9] and to planning conservation priorities [10–12], the use of this tool will provide inaccurate information when available data for species occurrence are very scarce (e.g., < 10 records of occurrence) [13,14]. In this situation, encompassing many threatened species, more field effort is then required to increase the empirical knowledge on species occurrence.

**Fig 1:**
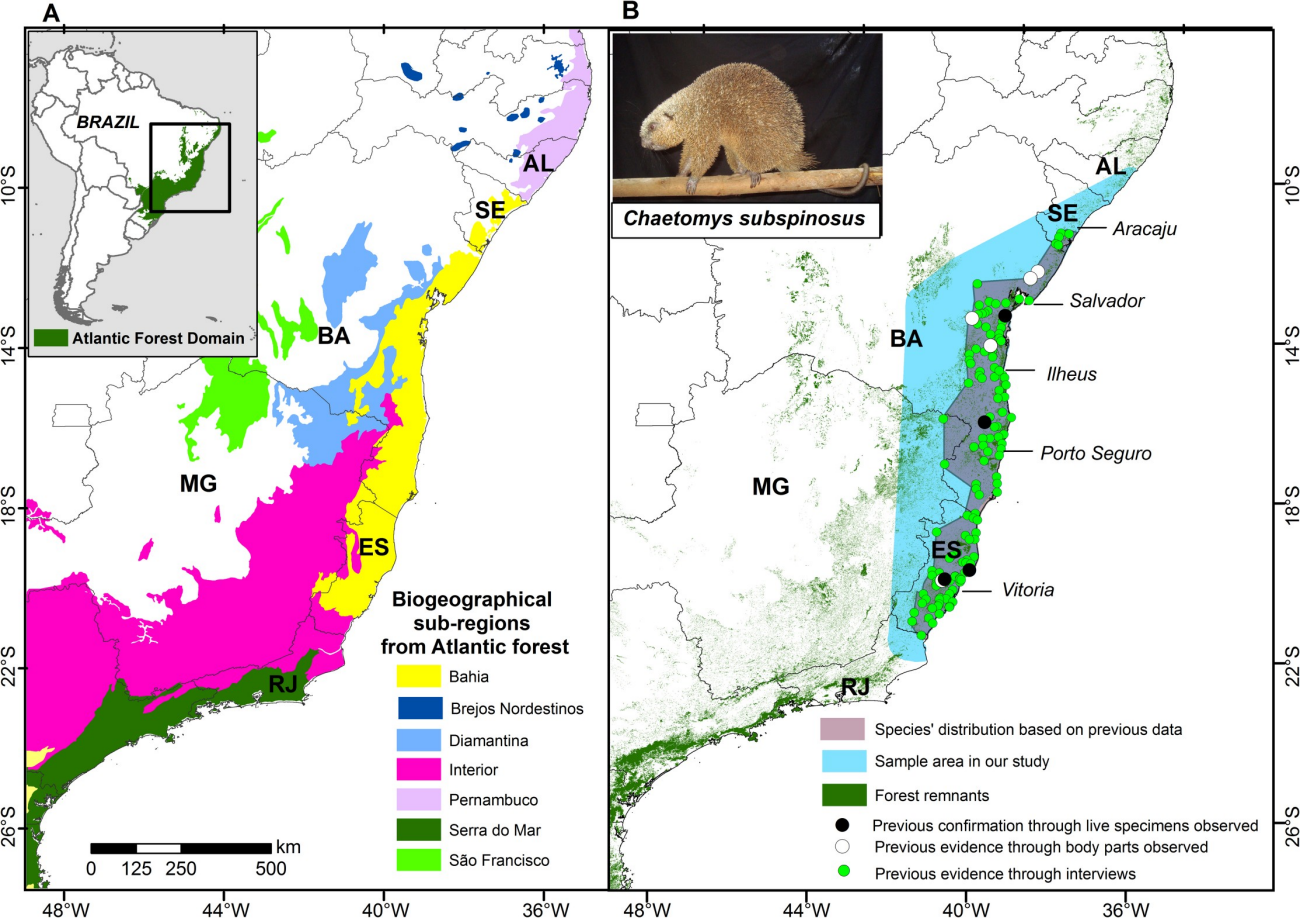
Map of the study area highlighting the biogeographic sub-regions of the Atlantic forest (A), the area sampled in our study and the geographic distribution of the thin-spined porcupine previously known through the study of Oliver and Santos [16] (B). Evidence data reported by these authors are presents in the map. Geographic Projection: Datum WGS 1984.

To date, this has been the case of the threatened thin-spined porcupine, *Chaetomys subspinosus* (Olfers 1818), an endemic mammal of the Atlantic forest of eastern Brazil, which was rarely detected in the wild during the 20^th^ century (< 10 confirmed records published) [15,16]. Its geographic distribution was previously investigated (Fig 1) based on interviews and few confirmed occurrences [16–18]. The species has been considered vulnerable to extinction since 1994 [19], and all assessments of its current conservation status, as well as conservation actions proposed, have been based on such unconfirmed mapping.

The thin-spined porcupine is a secretive forest porcupine (Family: Erethizontidae), strictly arboreal, folivorous and nocturnal [15,20–22], with high phylogenetic value as the single member of subfamily Chaetomyinae [23]. It is probably the most folivorous member of its family [20,22], and the most eurytopic and forest-dependent species among the erethizontids [23]. It is restricted to Atlantic forest, where ~82% of forest cover has been destroyed and fragmented [24], with remnants immersed in anthropogenic landscapes highly deforested and subject to constant human pressure [25]. Hunting and fire are some examples of such pressures [16], activities also occurring inside many protected areas [26]. This species is considered vulnerable to extinction by IUCN Red List due to its restricted occurrence as well as assessments suggesting a likely continued decline of its occupancy area, leading to a reduction in the quality and the number of subpopulations (criteria B1ab ii, iii and iv; 25). This species has been considered as a conservation priority because it is monotypic, endemic and often used by humans populations [26,27].

According to a previous study conducted in the 80's [16], the thin-spined porcupine is distributed in the central portion of the Atlantic forest from extreme northeastern corner of Rio de Janeiro (41^o^07'W 21^o^18'S) to southern Sergipe state (37^o^37'W 11^o^14'S), along the coast of Brazil, in the biogeographical sub-region known as "Bahia" (Fig 1). Particularly, two main hiatus were detected along its occurrence in this previous research [16]. The first one in the triangle immediately to the north of the municipality of Salvador (38^o^31'W 13^o^01'S), in the Bahia state, and the second and larger one in north-western parts of Espirito Santo (near to 40^o^13'W 18^o^24'S). This last hiatus finds further support, as populations from northern and southern are relatively divergent and genetically structured [28]. In addition, *Chaetomys subspinosus* occurs in sympatry and syntopy with the Bahia hairy dwarf porcupine, *Coendou insidiosus* (here named Bahia porcupine), which is considered more common, widely distributed and non-threatened [16]. It is unknown whether the *C*. *insidiosus* has an effect on the occurrence of the target species, but given the syntopy and phylogenetic proximity, these species may compete for resources [16,23].

As part of the activities of the National Action Plan (PAN) for the Conservation of this species [29], we conducted an extensive inventory searching for reports on species occurrence to provide a more reliable mapping and to produce a spatial model of environmental suitability and geographic distribution for the thin-spined porcupine, based on climatic and topographic conditions. Specifically, we combined species distribution modeling with field surveying to: (1) predict and validate the current geographical distribution of the species; (2) analyze the effect of the environmental suitability and forest cover on the relative likelihood of species incidence; (3) estimate the amount of suitable habitat in the species range and protected areas; (4) identify the most suitable larger forest fragments in protected and non-protected areas; and (5) indicate priorities for conservation and future research. Furthermore, we compared and related the incidence rate of the target-species with those of the sympatric *Coendou insidiosus*, aiming to offer a relative notion about the rarity and vulnerability of the thin-spined porcupine and to evaluate a potential effect of the occurrence of one over the other. We used the results of the interviews as an independent dataset to validate the predictions of the species distribution models, which were based on confirmed presence records of the species obtained by direct observations, and to calculate incidence rates. Our results provide a more comprehensive picture of the spatial distribution and vulnerability of the target species, paving the way to support more science-sound conservation decisions.

## Materials and methods

### Study area

We undertook a large-scale survey to check for the presence of the target species in the biogeographical sub-region (BSR) "Bahia" [30,31] and surrounding areas (Fig 1) from the central portion of the Atlantic forest, northeastern Brazil (36^o^01'-41^o^55'W and 10^o^02'-21^o^46'S). These limits included 212,843.64 km^2^ through the states of Bahia (BA), Espirito Santo (ES) and Sergipe (SE), and the neighboring parts of the states of Rio de Janeiro (RJ), Minas Gerais (MG), and Alagoas (AL), ranging 100 to 200 km beyond the species' geographical distribution previously suggested [16, 19]. The geographical limit of the survey was established when further advances no longer brought evidence of the species' presence during consecutive interviews and active searches.

The sub-region Bahia (Fig 1A) encompasses one of main centers of endemism along the Atlantic forest biome [30,31], where ~ 17.7% of the original native forest cover remains, composed mainly of small and isolated forest fragments, with great exposition to edge effects [24]. Protected areas included only ~ 4.2% of this remaining forest fragments [24]. The altitude varies between 0 m and 2376 m and the annual mean temperature range is between 10.9 and 25.6°C. Although most of the region is composed of lowland with warm temperature, some colder regions (annual mean temperature < 15°C) occur in higher elevations from southwestern of ES (reaching altitudes of 2376 m) and some sites from BA (reaching altitudes of 1090 m). The annual precipitation decreases from the coast to the interior, ranging between 2,402 and 553 mm.year^-1^, respectively. Due to this longitudinal rainfall gradient, evergreen forests, such as dense ombrophilous forests (lowland and montane tropical rain forests) and *restinga* formations (coastal sandbank vegetation) occur closer to the coast, while semi-deciduous and deciduous forests occur in the interior [32].

### Confirmed occurrence data

We compiled a database of 117 georeferenced localities of confirmed occurrences of the focal species (S1 Data), based on a direct observation of animals or body parts (quills, skins or bones) made by us (n = 50) and other researchers (n = 34) in the field, as well as including specimens registered in scientific collections (n = 33). We consulted the SpeciesLink platform (http://www.splink.org.br/) for obtaining data of scientific collections. We included data of direct observation provided by colleagues only when published or with the previous consent and species' identification confirmed by us through pictures. In order to increase our database and to find the target species through direct observation, we performed active field surveys during 571.5 hours, over 102 days, from 2004 to 2014, sampling 46 forest fragments located in 30 municipalities. For this, our fieldwork encompassed a 12,500 km round trip through of the study area. The active searches were conducted during the diurnal period by a team of two to three people including a researcher (G.A.F. Giné), an experienced field assistant and, on some occasions, a local guide. We walked inside forest remnants and visually inspected the intermediary and superior strata, mainly tangles of vines, forks, bromeliads, palms, and tree holes, which are structures commonly used by species as diurnal resting sites [16,21,33]. Once located, we captured, immobilized and photographed each animal and collected quills for genetic analysis [28,34]. Despite the effort directed, most of the records compiled in our database (75%) were results of opportunistic sightings, while 29 records were acquired through active search. Most (85%) of the data were obtained after 2004, while the others were obtained between 1986 and 2004. We conducted all procedures followed the guidelines of the American Society of Mammalogists [35] and under the legal approval and consent of the Brazilian Federal Authority (licenses number: 032/2004 CGFAU/LIC and 23468–1), which included permission to search and capture this species listed as Vulnerable on the IUCN Red List and on protected areas.

### Data from interviews

We created a database of 232 positive records of evidence of the local presence of the target species based on interviews, consisting of 119 records obtained by us and 113 reported in the literature [16]. For this, we conducted 236 interviews with local people from 122 municipalities along the study area. We conducted interviews from 2004 to 2014. We added data from 264 interviews from 144 municipalities provided by Oliver and Santos [16], which was collected in 1986 and 1987. Considering all the 500 interviews (S2 Data), we assessed the information of local knowledge of species occurrence from 215 municipalities. We did not apply this database to build the species distribution models (SDMs) but employed it as an independent dataset to evaluate the predictive power of the models. We performed interviews in locations separated by > 2 km, with rural residents locally identified as being the most knowledgeable regarding the wildlife in each area, in general hunters and people living near forest fragments (< 100m). We considered as positive evidence of local presence of the target species, when the interviewee met all the following requirements: they correctly described the species (based on external appearance, body weight, and quills; details in S1 File); recognized the species in a display of photos among several other mammal and porcupine species; recognized the quills of the species in a display case among quills from several erethizontid species; and claimed to have seen a specimen in the last five years in the locality. We applied the same method and recorded data about the sympatric *Coendou insidiosus* in order to calculate, compare and relate the incidence rates of this non-threatened species with those revealed by the target species. We discarded the interviews with dubious answers about the presence or absence of the species. Eventually, when the interviewee presented biological material of the species, we added such a record to the confirmed occurrence database and did not consider the interview data to avoid double use of the data. We followed a protocol similar to those applied by Oliver and Santos [16]. We previously clarified to respondents about the nature of the research and its objectives. The participation of the respondents, who were kept anonymous, was voluntary throughout the interview process and they agreed to contribute to the survey, providing verbal consent for the use of this information.

### Selection of the environmental variables for the modeling procedure

We downloaded 19 bioclimatic data sets from the WorldClim website (http://www.worldclim.org) [36] and 1 topographic data set (digital elevation model) developed by the U.S. Geological Survey EROS Data Center (GTOPO30; http://eros.usgs.gov/elevation-products; downloaded 20 March 2015) for the Atlantic forest region (34^o^11'-58^o^00'W and 2^o^04'-35^o^03'S), both with ~1 km spatial resolution (30 arc second). In order to evaluate and choose the most informative and non-redundant variables to predict species distribution, we previously tested the multicollinearity among all the variables by Pearson's correlation coefficients. Then, we submitted these 20 variables to MaxEnt Jackknife tests [37] using the software MaxEnt version 3.3.3 (http://www.cs.princeton.edu/~shapire/maxent/; downloaded 15 March 2008), following the same configuration used later for the modeling (see next topic). We ranked the 20 variables considering their contribution values obtained in the three Jackknife tests available in this software (Jackknife of training gain, test gain, and AUC). We selected the most informative variables, which had low correlation (r < 0.75) between them, and we check their biological sense for a final selection between highly correlated variables. We considered that drier periods and conditions may compromise the abundance and quality of tree leaves (source of food and water used by the thin-spined porcupine), thus reducing habitat suitability for this species, while conditions that influence higher primary productivity (hot and humid conditions) favor this species. We also considered that this species of arboreal folivores may be sensitive to a higher thermal variation and cold conditions due to energy constraints imposed by their diet and reduced body size. We therefore considered 8 environmental variables as potential predictors of the *C*. *subspinosus* distribution, namely: mean monthly temperature range (BIO2), isothermality (BIO3), maximum temperature of warmest month (BIO5), annual temperature range (BIO7), precipitation of driest month (BIO14), precipitation of wettest quarter (BIO16), precipitation of coldest quarter (BIO19) and elevation (ELV).

### Modeling procedure, species distribution and pattern of environmental suitability

We used 82 of 117 confirmed data of species presence to perform SDM analyses, as we considered only one record per pixel (~1 km^2^). We used random subsets composed of 80% and 20% of this dataset for training and test of the models, respectively. Given that each algorithm generates different forecasts and, due to the overall uncertain nature of species distribution models [38], we used three different algorithms to develop an ensemble potential geographic distributional model [39,40]. Algorithms used were: Bioclim (BIOCLIM) [41,42], Maximum Entropy (MaxEnt) [43] and Generalized Linear Model (GLM) [44]. We used the software MaxEnt version 3.3.3 to run MaxEnt algorithm and the *glm* (binomial distribution), *bioclim*, and *predict* functions from the *dismo* package to run the other two modeling algorithms in R software version 3.0.2 [45]. We performed ten replicates for each algorithm and used the mean suitability model of each algorithm for later analyses. In all model evaluations, we used 10,000 random pseudo-absences.

For each modeled suitability matrix, we generated a binary map based on the receiver operating characteristics (ROC) thresholds in order to determine "suitable" and "unsuitable" habitat. Such threshold method balances both omission and commission errors [46]. Then, we used these binary maps to produce a summed distribution map, and we considered the overlapping area from all models (consensual model) as the potential species distribution range. In addition, we created a second ensemble model with continuous values of environmental suitability. For this, we multiplied each modeled suitability matrix by the corresponding binary map separately, maintaining the continuous values above the threshold value while converting all values below the threshold to zero. Because many algorithms produce dimensionally different suitability ranges, we rescaled the outputs from each model to values of 0–1 by dividing each pixel by the maximum pixel value. Then, we averaged suitability values of the models creating a continuous ensemble suitability map. We used the ArcGIS natural breaks (Jenks) of the Spatial Analyst extension to define suitability categories. In this way, we delimited areas of very low (0 to 0.140), low (0.140 to 0.359), medium-low (0.359 to 0.558), medium-high (0.558 to 0.715) and high (0.715 to 1) suitability for target species. Then, we estimated the area of the potential species distribution and the extent of the zone of the high suitability (suitability values > 0.715) for this species.

Finally, we evaluated the predictive ability of the ensemble binary model considering the value of the area under the curve (AUC) obtained by receiver operating characteristic (ROC) plot method [47] and the True Skill Statistics under the ROC threshold (TSS_roc_) [48]. We evaluated this predictive ability using three modeling-independent datasets: the first composed of 20% of randomly selected confirmed occurrence locations, the second composed of 232 positive evidence records obtained through all interviews and the third composed of 119 positive evidence records obtained from interviews conducted by us after the year 2004.

### Remaining habitat, protected and “stronghold” areas

Based on the current map available for the forest cover remnants of the Atlantic forest from *Fundação SOS Mata Atlântica* [49], we estimated the total area of the suitable forest included in the species range. We assumed that native forest and arboreal *restinga* (Brazilian sandbank forest vegetation) included in its distribution are the unique suitable habitats for the focal species, as suggested in the current literature [25,50]. Also, we classified and quantified the forested habitats according to three vegetation types defined by digital Brazilian Vegetation Map [51]: dense rainforest or evergreen ombrophilous forest; *restinga*; and seasonal forest (deciduous and semi-deciduous forest).

We identified the protected areas included in species geographical distribution using a digital map of the Federal and State Brazilian Nature Reserves (information from CNUC/MMA http://mapas.mma.gov.br/i3geo/datadownload.htm) classified as ‘‘proteção integral” which is equivalent to the IUCN categories Ia and II [52]. We further listed the protected areas in which the presence of the target species was confirmed. By superimposing the maps of forest cover remnants, ensemble suitability, and the current reserve system, we estimated the total forested area under protection and identified protected and non-protected stronghold areas, i.e., the larger forest fragments in high-suitability zones.

### Factors influencing species incidence

We evaluated the importance of the climatic suitability and the remaining forest cover at a regional scale on the proportion of positive evidence of the presence of the focal species obtained by interviews, herein defined as species incidence rate. We assumed that this response variable is a measure of how common the species is in each region. Firstly, we superposed a hexagonal grid of cell size equal to 5000 km^2^ over the sampled area and, for each hexagon cell, we calculated the proportion of the positive interviews, the mean climatic suitability and the percentage of the forest cover. We only considered for analyses the hexagonal cells with five or more interviews, totaling 36 cells. Because previous analysis indicated a low correlation between mean climatic suitability and the percentage of the forest cover (Pearson's correlation; r = 0.46), we included both variables in the model tests. We performed Piecewise and General Linear Models (GLM), defined by all possible variable combinations and the null models, with a binomial distribution, log link functions and weighted by the number of interviews obtained in each hexagonal cell. Then, we selected the most parsimonious model after ranking the candidates based on Akaike's Information Criterion corrected for small sample size (AICc). Models were considered equally plausible when AICc differences (Δi) were lower than 2 [53]. When the piecewise models were selected as best models, we tested the significance of the breakpoint by the Davies' test [54] using the “davies.test” function from “segmented” package. For comparison purposes, we also evaluated the influence of forest loss on the incidence rate of the non-threatened porcupine, *Coendou insidiosus*, by building a binomial GLM (weighted by the number of interviews) and contrasting this with null model based on AICc. Furthermore, we performed a Pearson's correlation between the incidence rates of the two porcupine species to evaluate the effects of the incidence rate of one over the other. We processed all spatial data using ArcGIS version 9.2 software [55] and performed statistical analysis using the R software [45].

## Results

### Model evaluation

Based on confirmed records, the ensemble model showed high values of AUC and TSS_roc_ (0.995 and 0.960, respectively), indicating a potential high predictive performance. Also, based on all interview records (232 locations) and those performed after 2004 (119 locations), the ensemble model also showed high AUC (0.964 and 0.973, respectively) and TSS_roc_ values (0.88 and 0.91), validating such performance.

### Species distribution and pattern of environmental suitability

**Fig 2:**
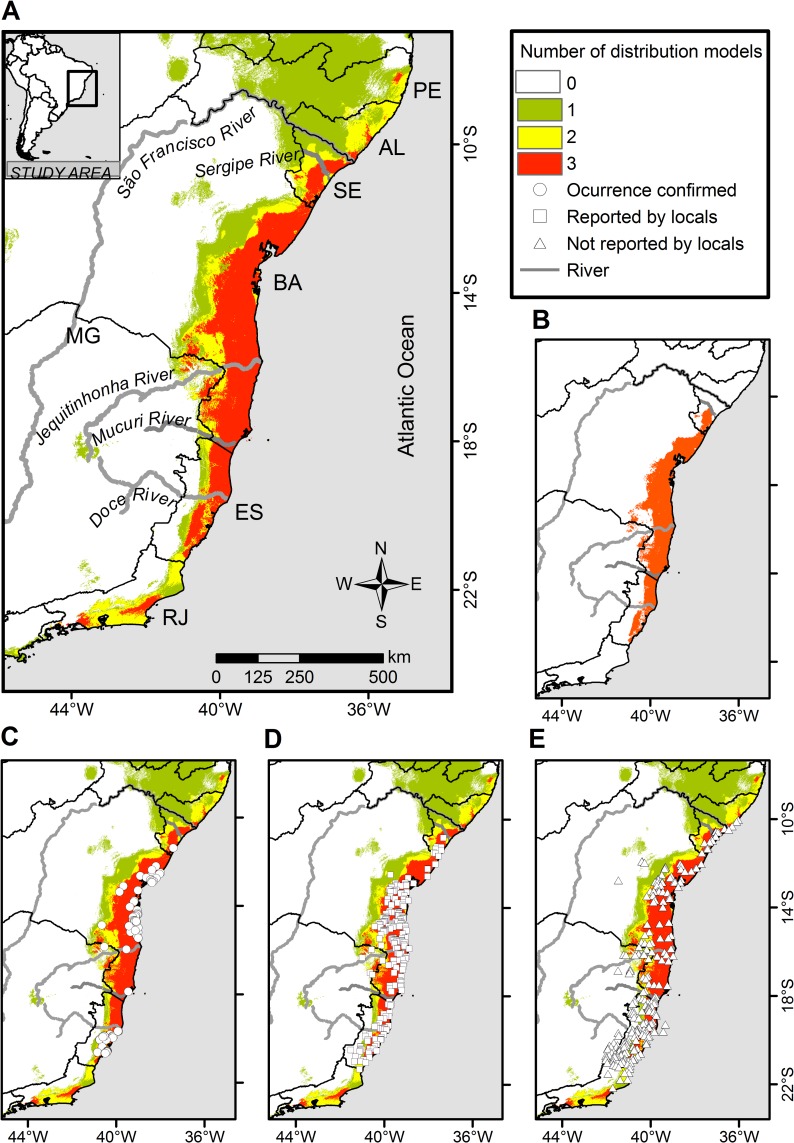
Potential geographic distribution of the thin-spined porcupine (*Chaetomys subspinosus*) in the Atlantic Forest, Brazil, based on the binary ensemble model of three algorithms: Bioclim, Maxent and Generalized Linear Model (GLM). The binary ensemble model is shown by the overlap of three models (A). The most reliable distribution based on the ensemble model and survey empirical data are shown in the map (B). The confirmed occurrence points used in the modeling are also shown in the map (C), as well as the sites where the target species was reported (D) and not reported (E) by locals during interviews. Geographic Projection: Datum WGS 1984.

The binary ensemble model (Fig 2A) predicted suitable conditions for the thin-spined porcupine in the eastern part of ES, BA, and SE states, reaching to some discontinuous areas from AL, MG and RJ states, comprising a total area of 112,133 km^2^. However, our field efforts indicated that, although there is climate suitability, the focal species was unambiguously not reported along the coast of the states of RJ, AL and northeastern SE (further north to Sergipe river; Fig 2D and 2E). Notably, no porcupine species were known in northeastern SE state, and only *Coendou prehensilis* was reported in the states of AL and PE. Therefore, our field efforts indicated that the species is distributed between the extreme southeastern corner of ES (21^o^10`S; 40^o^54`W) to central-eastern SE (10^o^46`S; 37^o^18`W), along the coast of the Atlantic forest, including the states of BA, and the extreme northeastern corner of MG (Fig 2B). Excluding all the northern and southern extreme portions (RJ, AL states, and northern Sergipe river), which are small areas where model commissions (false positives) apparently occurred, the estimated extent of occurrence of the target species totaled 104,326 km^2^ (Fig 2B). We used this range for subsequent analysis, as this comprised the most reliable picture of the current realized species distribution, although we also present in the Supplemental Material (S1 Table) the quantitative attributes of the entire area predicted as environmentally suitable for the species.

**Fig 3:**
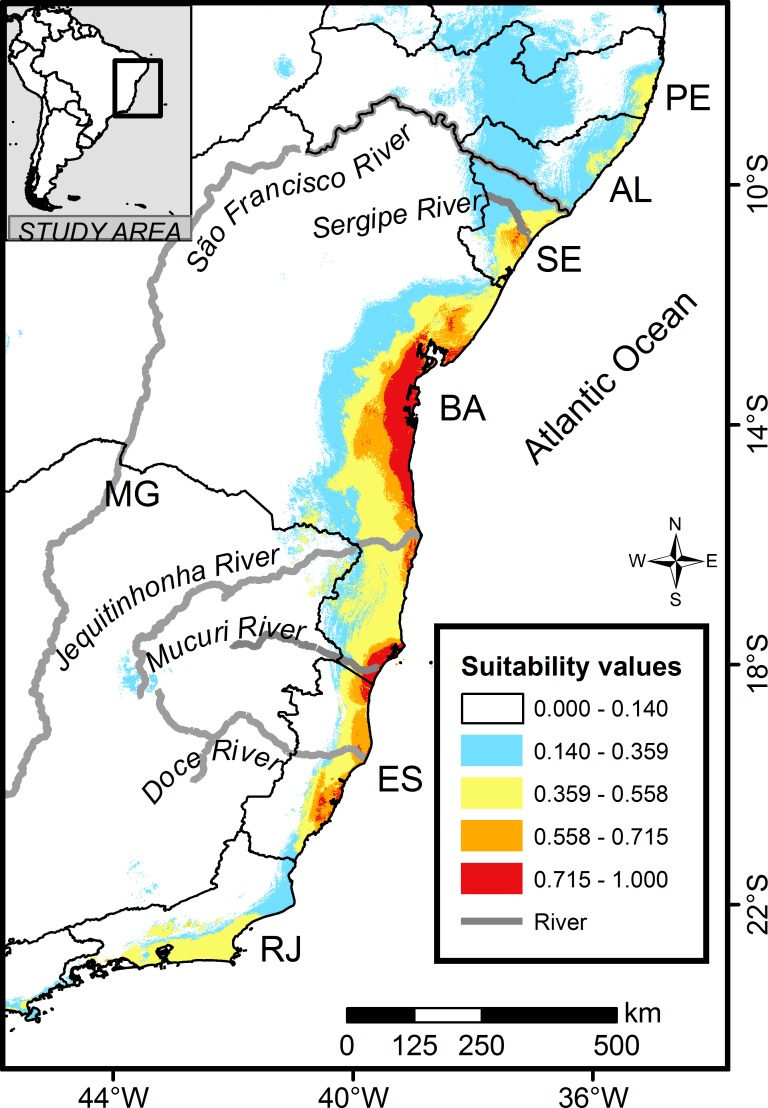
Predicted environmental suitability for the thin-spined porcupine (*Chaetomys subspinosus*) in the Atlantic Forest, Brazil, based on continuous ensemble values estimated through of three algorithms: Bioclim, Maxent and Generalized Linear Model (GLM). Ensemble suitability values are the average suitability of rescaled values produced by three algorithms. Suitability categories were defined using ArcGis natural breaks (Jenks) as: very low (0 to 0.140), low (0.140 to 0.359), medium-low (0.359 to 0.558), medium-high (0.558 to 0.715) and high suitability (0.715 to 1). Geographic Projection: Datum WGS 1984.

Our continuous ensemble suitability model (Fig 3) predicted high suitability areas (importance value ≥ 71.5%) covering approximately 16.1% of the potential area of occurrence, totaling 16,813 km^2^. We identified two main continuous areas of high suitability. The first and more extensive one was located along the coast of BA (south-north), between the municipalities of Canavieiras (southeastern BA) and Santo Amaro (metropolitan region of Salvador, BA), into some parts stretching up to 60 km to the west. The second area reaches from the extreme northern ES (Conceição da Barra) to the south of the BA (municipality of Caravelas), ranging up to 25 km to the interior. In general, environmental suitability was decreasing from the coast towards the interior (west) throughout the estimated occurrence and, mainly, was lower (importance value < 55.8%) in the RJ, AL, MG states and some coastal areas from southeastern and northeastern ES and BA states.

### Remaining habitat, protected and “strongholds” areas

**Fig 4:**
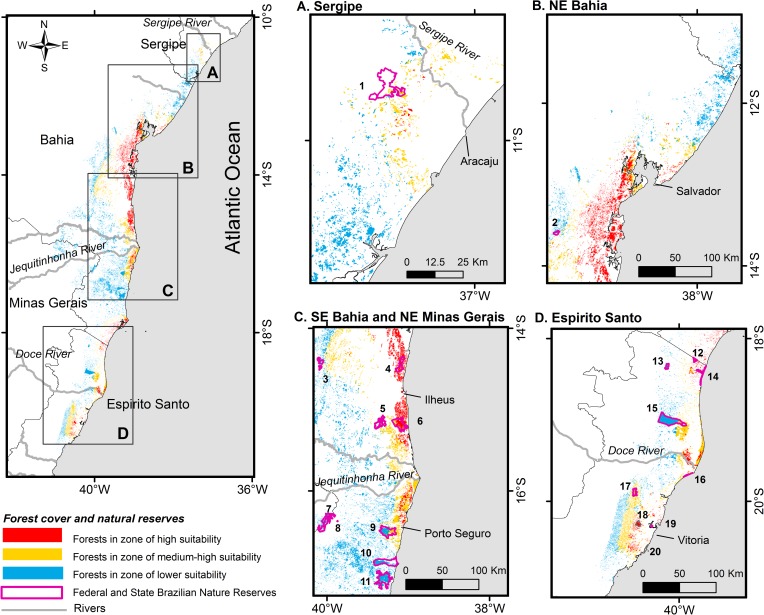
Protected areas and remaining Atlantic forest in the potential geographical distribution of the thin-spined porcupine (*Chaetomys subspinosus*) differentiated according to class of the environmental suitability predicted by modeling procedures, which are: lower (0 to 0.558), medium-high (0.558 to 0.715) and high suitability (0.715 to 1). In detail, the following regions are shown: (A) Sergipe state; (B) northeast of Bahia; (C) southeast of Bahia and northeast of Minas Gerais; and (D) Espirito Santo state. Protected areas are numbered and can be identified following Table 1.

According to our estimates, 13.3% of the original Atlantic forest remains within the species potential distribution (Fig 4). This forest cover is composed of 22,476 forest fragments covering 13,870 km^2^. We estimated that 80.9% of the fragments is represented by small fragments (<50 ha), followed by medium (50 to 250 ha; 15.5%), medium-large (250 to 1000 ha; 3%) and large fragments (>1000 ha; 0.6%). In the zone of high suitability for the target species, 3,299 km^2^ of forest cover remains, most concentrated in the southern and center-eastern BA state and classified as ombrophilous forest (82.8%) and restinga (16.7%). More details on the profile of the forest remaining in the suitable and highly-suitable range are presented in S1 Table.

We identified 20 federal and state protected areas within the potential geographic distribution of focal species (Table 1; Fig 4), covering 1,177 km^2^ of forest, i.e., 8.5% of the total forest cover present in the species range. The three largest fragments along the potential occurrence range of the target species are entirely or partially included within the limits of three protected areas: Descobrimento National Park (BA) (240.7 km^2^), Sooretama Biological Reserve (ES) (233.0 km^2^) and Pau-Brasil National Park (BA) (218.8 km^2^). However, these vast reserves are located outside the predicted high-suitability zones. Only 207 km^2^ of the protected forest was estimated within high-suitability zones (suitability value ≥ 71.5%), and it is represented by seven reserves (Table 1). Among these, the most extensive protected forests include the Una Biological Reserve (BA) (124.3 km^2^), Serra do Conduru State Park (BA) (66.6 km^2^) and Córrego Grande Biological Reserve (ES) (12.7 km^2^). The presence of the focal species was confirmed in seven reserves (Table 1), totaling 523 km^2^ of legally protected habitats.

**Table 1:** Federal and state protected areas in the potential geographical distribution of the thin-spined porcupine (*Chaetomys subspinosus*) and the total of protected forest area in the predicted suitable and high-suitability climatic zone for the species.

	Protected area	State	Forest area (ha)	High-suitability forest area (ha)
1	Parque Nacional da Serra de Itabaiana	SE	1031	72
2	Estação Ecológica Estadual Wenceslau Guimarães	BA	979	0
3	Parque Nacional de Boa Nova	BA	2240	0
4	Parque Estadual da Serra do Conduru** [50]	BA	6663	6663
5	Parque Nacional da Serra das Lontras *	BA	1609	0
6	Reserva Biológica de Una** [50]	BA	14264	12429
7	Parque Estadual Alto do Cariri	MG	3000	0
8	Parque Nacional do Alto Cariri	MG	5016	0
9	Parque Nacional Pau Brasil	BA	16312	0
10	Parque Nacional do Monte Pascoal	BA	11958	0
11	Parque Nacional do Descobrimento	BA	18194	0
12	Reserva Biológica do Córrego Grande	ES	1268	1268
13	Reserva Biológica do Córrego Do Veado	ES	2215	0
14	Parque Estadual de Itaúnas	ES	267	166
15	Reserva Biológica de Sooretama** [56, 57]	ES	25823	0
16	Reserva Biológica de Comboios *	ES	298	48
17	Reserva Biológica Augusto Ruschi** [16]	ES	3185	0
18	Reserva Biológica de Duas Bocas	ES	2847	0
19	Parque Estadual da Fonte Grande	ES	72	52
20	Parque Estadual Paulo César Vinha** [22, 33]	ES	422	0
	Total area		117663	20698

* Protected areas where the presence of the species was confirmed by us through photo

** Protected areas where the presence of the species was recorded in the scientific literature.

Outside protected areas, we identified three large blocks of forests in the zone of high suitability, each comprising two large fragments located along the coast of BA (Fig 4B and 4C). The largest block was located within the municipalities of the Maragogipe (136.6 km^2^) and Cachoeira (101.2 km^2^), 30 km to south of the Salvador (38^o^42'-38^o^56'W and 12^o^41'-12^o^57'S); the second includes the municipalities Santa Cruz de Cabrália (120.3 km^2^) and Belmonte (114.9 km^2^), 25 km to north of the Porto Seguro-BA (38^o^58'-39^o^10'W and 16^o^01'-16^o^14'S); and the third block was located in Nilo Peçanha (80.3 km^2^) and Cairu (20.3 km^2^) municipalities, 80 km to south of the Salvador (39^o^00'-39^o^06'W and 13^o^30'-13^o^43'S).

### Factors influencing species incidence

**Fig 5:**
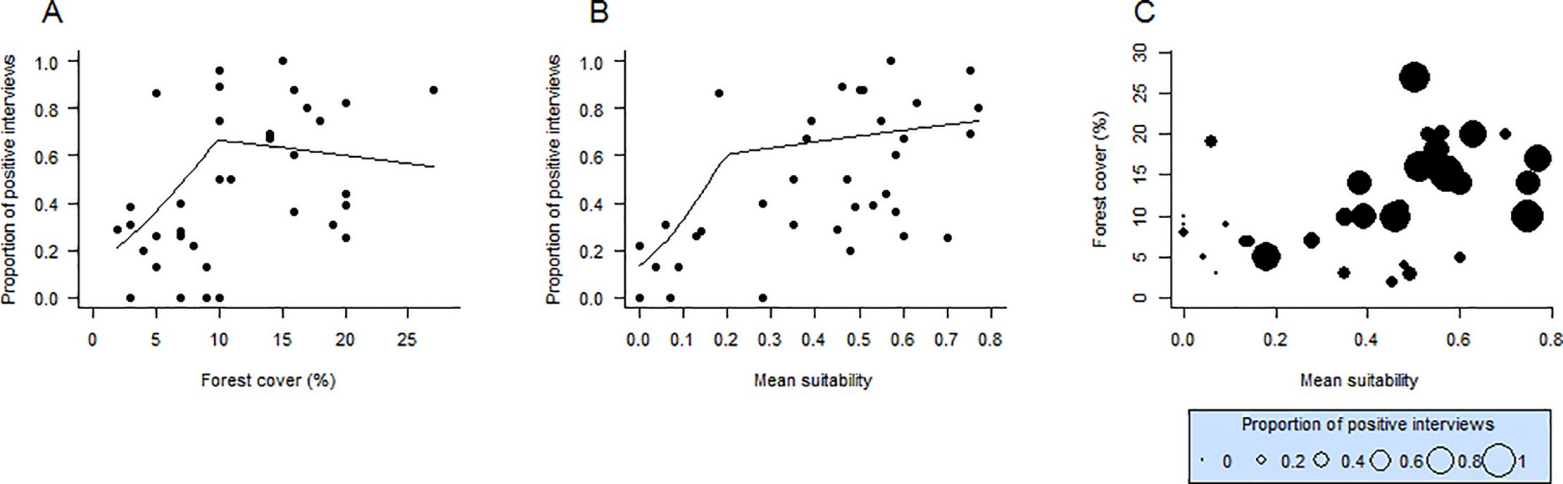
Influence of the percentage of forest cover (A) and mean environmental suitability (B) on the proportion of the interviews in which the thin-spined porcupine (*Chaetomys subspinosus*) was reported by locals (herein proportion of positive interviews) in different regions of the species’ potential geographical distribution. The relationships between these three variables are shown in the bubble chart (C), where the size of the bubble represents the percentage of positive interviews in each region. Regions (n = 36) were delimited overlapping a hexagon grid of the cell size equal to 5000 km^2^ on the species’ geographical distributions. Only cells with five or more interviews were considered. The line with a breakpoint represents the curve adjusted for the piecewise relation identified according to the “better” model shown in Table 2.

**Fig 6:**
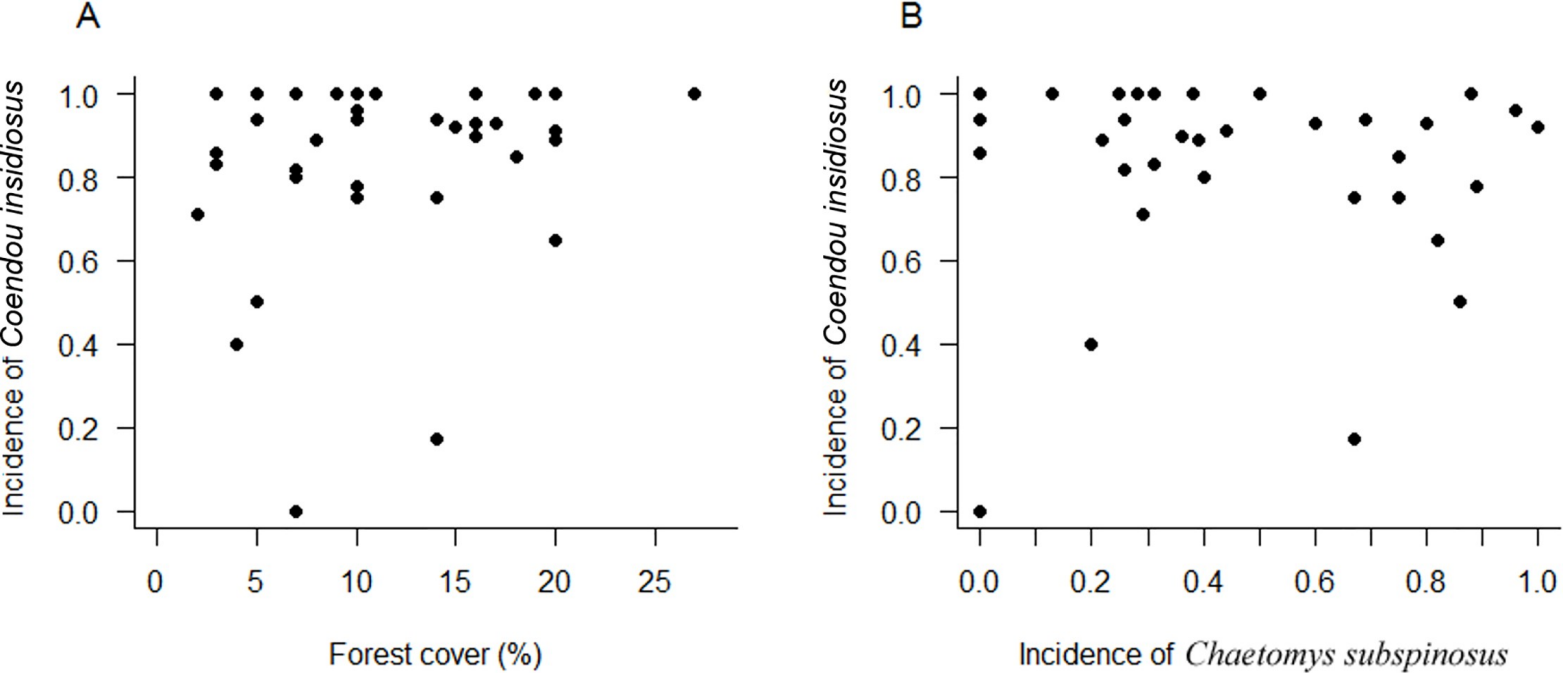
Relationship between the percentage of forest cover and the proportion of the interviews in which the non-threatened Bahia porcupine (*Coendou insidiosus*) was reported by locals (herein named incidence rate) in different regions of the *Chaetomys*’ potential geographical distribution (A). Relationship between the rate of incidence of *Coendou insidiosus* and *Chaetomys subspinosus* (B). Regions (n = 36) were delimited overlapping a hexagon grid of the cell size equal to 5000 km^2^ on the species’ geographical distributions. Only cells with five or more interviews were considered in the analysis.

The occurrence of the thin-spined porcupines (*Chaetomys subspinosus*) was detected in 46.51% of the 500 interviews performed. Specifically, this species was detected in 50.4% of the interviews performed by us and 43.0% those performed in the 1980s [16]. The piecewise model including environmental suitability and forest cover as predictor variables was the best model explaining species incidence rate among those tested (Table 2), indicating that both variables influence the variation of how much commonly the species is known by locals in the different regions along its distribution. The breakpoint (Fig 5) indicated that the proportion of the positive interviews was significantly and abruptly reduced when forest cover (within a 5.000 km^2^ scale) was lower than 10% (SE = 1.57, n = 36, *p* = 0.006) and mean suitability was lower than 0.20 (SE = 0.06, n = 36, *p* = 0.036). Even in regions with medium-high environmental suitability, there was a low incidence rate of the species when forest cover was lower than 10% (Fig 5C). Such levels of the deforestation (< 10%) tended to occur in western, southern and northern extremes of the distributional range (southern ES and northern SE), and between northeastern ES and southeastern BA (Fig 4). In comparison, the Bahia porcupine (*Coendou insidiosus*) was detected in 87.7% of the 500 interviews. The incidence rate of this non-threatened species was not significantly influenced by percentage of forest cover, once the null model was equally plausible for explain the incidence variation (Δi = 1.2) according to AICc (AICc = 172.70, df = 2, ωi = 0.64; AICc = 173.9, df = 1, ωi = 0.36, respectively). In general, a high incidence rate of this species occurred even in regions with low forest cover (Fig 6A). Finally, the rate of incidence of the two porcupine species was not correlated (r = 0.02, df = 34, *p* = 0.890, Fig 6B).

**Table 2:** Akaike (AIC) based model selection for the proportion of positive records of evidence of the presence of thin-spined porcupines (*Chaetomys subspinosus*) in landscapes (hexagons = 5000 km^2^) from Atlantic forest obtained by interviews with locals along the species’ extent of occurrence. Piecewise and Generalized Linear Models (GLM) used the mean environmental suitability predicted by species distribution modeling and percentage of the forest cover as fixed factors and weighted the response variable by the number of the interviews. We also show the number of degrees of freedom (Df), AIC differences (Δi), and Akaike weights (ωi).

Candidate models	Df	AIC	Δi	Ωi
Piecewise: mean suitability + forest cover	7	188.86	0	0.990
GLM: mean suitability + forest cover	3	198.85	10.0	0.007
GLM: mean suitability	2	201.85	13.0	0.001
Piecewise: mean suitability	4	201.91	13.1	0.001
Piecewise: forest cover	4	240.59	51.7	<0.001
GLM: forest cover	2	249.74	60.9	<0.001
GLM: null model	1	272.84	84.0	<0.001

## Discussion

### Species distribution

This is the first study evaluating the geographic distribution of the thin-spined porcupine based on the most updated dataset of confirmed records and using species distribution modeling methods. Our effort allowed a considerable increase in the database of species occurrence locations and indicated the presence of the species between the extreme southeastern corner of the Espirito Santo and central-eastern of the Sergipe state, along the coast of the Atlantic forest. Our ensemble distribution model showed good predictive accuracy (AUC and TSS values > 0.95) suggesting that, in general, the environmental variables were important determinants of the species distribution in the Atlantic Forest. However, our field survey indicated that the binary model was not accurate in its entire extent, mainly at the northern boundaries of the estimated distribution. Other features not accounted in our modeling, such biogeographical barriers, ecological interactions, habitat availability, and human disturbance, may have influenced the northern boundaries of the realized species distribution, generating a small-extent noise not predicted by the model [58]. Thus, a careful interpretation of the binary model is required, since such model represents the potential suitable space based on coarse-grain climate and topographic variables, i.e., a fundamental grinnellian niche [59,60]. On another hand, the interview data were useful to recognize the commission areas of the model and to adjust it empirically, resulting in the delimitation of a potential realized distribution. Once such model commissions were identified in small and deforested regions, there were no abrupt changes in the overall quantitative results obtained from this adjusted discrete model, and we provide information of both models (predict and adjusted) to inform uncertainties and better guide conservation decisions (see S1 Table). In addition, we warned that the models based on presence-only data have limitations since they assume that the detectability is constant in space, not allowing to distinguish the species occurrence from its detection probabilities [61]. It was not possible to include the imperfect detection in our model since the majority (75%) of the occurrence data available were results of opportunistic observations (opportunistic sightings, scientific collection and literature data). Despite such limitations, our continuous model was based on the best knowledge we have about the occurrence of this vulnerable species so far, revealing an approximate environmental suitability gradient for this and being potentially useful for the conservation management since it allowed identifying the potential strongholds areas for their protection.

Our predicted species distribution is similar to the first geographical distribution range suggested by Oliver and Santos [16], which was mainly based on interview data. However, we highlighted differences regarding the southern and northern boundaries previously suggested. First, even with our considerable sampling effort, including active search, interviews, literature, and information from experts, there was no evidence of the species current presence in northern RJ state, where our ensemble models predicted unsuitable climatic conditions for the target species. We, therefore, suggest that southern ES, and not RJ state should be considered the current southernmost limit of species distribution, while no evidence is confirmed for this state. Secondly, we confirm the presence of the target species in southeastern part of SE (Fazenda Crasto, municipality of Santa Luzia do Itanhy-SE), corresponding to the northern limit of the species range previously suggested [16], and we provide evidence that species distribution extends to the central region of this state (10^o^46`S; 37^o^18`W). Although the ensemble model indicated suitable environmental conditions farther north, in northeastern SE and some eastern regions of AL and PE states, the interviewees indicated no evidence of the species occurrence in these regions. The original northern boundary of its distribution is still unknown, and we do not discount a possible distribution retreat due to the high deforestation of these northern regions, currently with less than 5% of the original forest cover remaining. Despite the likelihood of its past occurrence, it is now clear that the species is no longer present in north of the Sergipe River. Although more research in this state is necessary, we suggest that this river from central-eastern of SE is potentially the northern boundary of its current distribution.

Our results do not support the idea for a distributional hiatus “in the triangle immediately to the north of municipality of Salvador-BA” as suggested by Oliver and Santos [16], as we confirmed the species presence in the metropolitan area of Salvador and surrounding municipalities. By contrast, our data are consistent with the hiatus encompassing the north-central and north-western part of the ES [16], where none presence of the target species was detected, even in protected areas with suitable environmental conditions (e.g., Córrego do Veado Biological Reserve). Near this region, the interviews indicated that the target species is present only in northeastern ES, restricted to a narrow strip along the coast (< 15 km of the sea), where the ensemble model predicted a high-suitability zone. Our analysis suggested that the current low incidence of this species (reported in 26.1% of the interviews) in this high-suitability zone (60.2%) may be due to low remaining forest cover (5.2%), as discussed later.

### Environmental suitability, deforestation, and species incidence

The predictive accuracy of the ensemble distribution model suggests that the environmental variables considered were good determinants of the species distribution. Lower environmental suitability was predicted mainly in the western region of the Atlantic forest, which is the driest region dominated by seasonal forests (deciduous and semi-deciduous forests). This gradient is largely consistent with expectations based on the ecological needs of this folivore mammal species. Considering that leaves are the primary nutritional and water source for the thin-spined porcupine [20,22], driest and seasonal conditions may influence habitat suitability due to discontinuous or insufficient availability and quality of tree leaves throughout the year. As the most folivore arboreal mammals are usually associated with evergreen forests [62], it is only reasonable that the perennial ombrophilous forests are the core habitat for the target species along the Atlantic forest.

In our interviews, we detected the presence of thin-spined porcupines in half (50.4%) of our sampling locations, similar to the rate obtained in the 1980s (44%) [16]. Although no significant change was observed in the percentage of positive records or in the extent of occurrence during ~20 years, in both assessments the target species was considered as extinct or at least so rare that it had not been detected in approximately half of the sampling locations. Comparatively, the sympatric Bahia porcupine, *Coendou insidiosus*, was detected in 89.5% of the sampling locations during our field effort and 87% of the locations investigated in the 1980s [16], suggesting that *Chaetomys subspinosus* is rarer or absent in localities where *Coendou insidiosus* persists. It is possible that the thin-spined porcupine to remain unnoticed and more secretive because they are less adaptable to human habitat [21,26,50]. However, we conducted the interviews preferentially with residents integrated with local wildlife and forest remnants, which were locally identified as being the most knowledgeable regarding the wildlife in each area, in order to minimize omission errors, i.e., the chances of the species being present and not being reported. Although *Coendou insidiosus* may be a potential competitor of *Chaetomys subspinosus* in the forest fragments, our analysis based on a broader spatial scale showed that there is no clear relationship between the incidence rate of these two porcupine species (Fig 6B) as well as there is no published information so far on resource sharing and competition between them. However, further studies must be conducted to investigate this subject.

In addition to revealing that the target species is rarer than *C*. *insidiosus*, we offer evidence that the incidence rate of *Chaetomys* along its species range varies with regional climate and forest cover. The match of environmental suitability and the presence of a minimum forest cover remaining within a regional scale (5,000 km^2^) were two important factors explaining regional species incidence. In general, the target species tended to be rarer in western parts of its distribution, even in regions where seasonal forests are still relatively well represented (i.e., high forest cover remains), likely due to the low environmental suitability as discussed earlier. On the other hand, higher climate suitability was not sufficient to determine species incidence rate, as it becomes abruptly rarer when the forest cover reaches values lower than 10% within a region, even with medium-high environmental suitability. Habitat loss and fragmentation often negatively affect the persistence of forest specialist mammals [63], which seems the case of the thin-spined porcupine but not of the Bahia porcupine. These results reinforce the idea that *Chaetomys subspinosus* is most eurytopic and forest-dependent than *Coendou insidiosus*. Even with biological features that indicate a high capacity of persistence in small fragments, such as small home ranges [33,50] and diet based on leaves of the abundant pioneer trees [20], the landscape scale habitat loss has apparently affected the populations of the thin-spined porcupine. Due to its dependence on native forests [50], deforestation and threats associated must result in a demographic decline. We offer here a proxy of a habitat loss threshold (~10%) for the species persistence in a wide scale (5000 km^2^). Considering that only 13.3% of the original forest cover remains within the species distribution area, our result suggests an alarming scenario for species conservation.

### Suitable forest fragments areas, protected areas, and conservation recommendations

Our study has shown the target species has experienced an impressive shrinkage of its original forest habitat, being rare in many poorly forested regions of its distribution. This situation may be aggravated, considering that 80% of the forests remnants consist of small fragments (< 50 ha), which can be lost in the coming years. Besides, recent estimates indicate that deforestation has been increasing in later years mainly in the region of higher suitability for this species [64]. For example, among 2015 and 2016 the Bahia state lost approximately 123 km^2^ of its forest cover, with the highest deforested area (~51 km^2^) occurring on the second largest non-protected strongholds here considered for the species (Santa Cruz Cabrália and Belmonte municipalities). Bahia state stood out to be the one that deforested more in this period [64], a worrying scenario for species conservation. The impact associated with human growth and an incompatible developmental policy has degraded and brought the prospect of an even greater loss of habitat in this biome. Brazilian environmental legislation and policies have proved fragile for the conservation of biodiversity in the long term and outside of the Natural Strict reserves [65]. In this context, although the conservation and restoration of forest fragments outside protected areas are needed and important initiatives in conservation schemes designed for this species, so far strictly protected areas have been the most solid foundation for their conservation.

We estimated that 8.5% of all existing forest in the current species range is included within 20 federal and state protected areas and we confirmed the species presence in seven of these reserves, which include 3.8% of all remaining forest. Thus, the conservation scenario of the thin-spined porcupine is not altogether pessimistic, considering the low spatial requirement of each individual (mean home range size of ~ 2 ha) [33,50,66]. However, to date, there is no knowledge about population data such as occupancy pattern, population density, growth rates, spatial organization, and dispersal ability. Studies about such issues are necessary in order to have a more precise conclusion about the size and viability of the population being protected in these areas.

Of particular importance, here we have identified the largest forest fragments in the high-suitability climatic zone, herein named “stronghold areas”, which are likely to represent important sites to accommodate larger populations of this species. In general, the stronghold areas were concentrated in coastal regions of southern and center-east of BA (Fig 4B and 4C), a region that is already considered a high priority for conservation [27] but continues to be severely impacted. In particular, we identified three non-protected strongholds areas along the coast of BA, to which we recommend special attention, given its dimensions (>100 km^2^ each) and current human pressures. Besides, only 6.3% of forest area from the high-suitability zone is currently protected, and two protected reserves represent 92.2% of this area: the Una Biological Reserve and Serra do Conduru State Park. These results highlights a worrying situation of low representation and poor distribution of protected areas within the optimal climatic regions predicted for the focal species. Moreover, even within these two large protected areas, hunting, the use of fire and deforestation are frequent anthropogenic pressures threatening the survival of the thin-spined porcupines [26]. Unfortunately, both protected areas are still being expropriated by the Brazilian government, as well as the limited number of technical staff to monitor and curb illegal activities carried out by the residents still living inside the reserves is a severe problem to be solved.

Finally, although species presence should be firstly confirmed, the Córrego Grande Biological Reserve and State Park of Itaúnas in northeastern ES are probably two important conservation units to be considered in any spatial prioritization. Both areas are part of a narrow strip of high climate suitability, which potentially has an important function of connecting populations. This region from northeastern ES is highly deforested (5.2%) and threatened, mainly due to eucalyptus plantations and tourist developments. Therefore, we recommended actions to maintain the integrity of these protected areas and to preserve and restore forests in this region.

### Conservation status and conclusions

Combining species distribution modeling and field survey, our study brings an updated picture of the potential distribution of a high priority species currently under threat, identifying some critical areas for conservation and future assessments that could guide decision makers. Although our survey indicates that the extent of species geographic distribution did not suffer a significant retraction, it is clear that habitat loss is a significant driver determining species decline within its extent of occurrence. Our results suggest that *Chaetomys* populations are sensitive to habitat loss and the high deforestation level of the Atlantic forest is already close to the limit of regional species resistance. The target species has already lost more than 86% of its habitat, and the most extensive stronghold areas for this species are currently on low protection and progressive deforestation. This information together with the fact that hunting and fire are pervasive pressures, also taking place within some protected areas that are key for the species conservation, support the notion that the target species is currently under threat.

We suggest that any further reduction in the current forest cover from Atlantic forest should be a major concern for conservation purposes. We agree that avoidance of deforestation is a major challenge faced by conservationists in the Atlantic rainforest, particularly after the recent federal law (Brazilian Forest Code, Law 12,727 of 17 October 2012) allowing further deforestation of native habitats along the entire Brazilian territory [65]. We hope that our models can help decision makers to more adequately prioritize their actions, including the identification of key regions, conservation units, and stronghold areas, but also to address how to curb hunting and fire events triggered by human presence. Future research to fill the gaps in knowledge about population issues such as density, occupancy patterns, and growth rates is encouraged. Later a population viability analysis for the species should be prioritized in order to help decision-makers to achieve the task of conserving the target species.

## Supporting information

S1 Data Georeferenced localities of confirmed occurrences of the thin-spined porcupine (*Chaetomys subspinosus*) based on direct observations and specimens registered in scientific collections. Direct observation data provided by colleagues were included only with the previous consent and species' identification confirmed by us through pictures.
(XLS)

S2 Data Georeferenced localities of positive and negative evidence of the presence of the thin-spined porcupine (*Chaetomys subspinosus*) based on interviews with locals. (XLS)

S1 File Differences between sympatric and allopatric porcupines from the Central Atlantic forest based on body weight, quills, and external appearance. (PDF)

S1 Table Characterization of the remaining Atlantic forest within the potential extent of occurrence of the thin-spined porcupine (*Chaetomys subspinosus*) and zone of high climatic suitability predicted by modeling procedures. (DOCX)
