# Supplementary material for: Combining species distribution modeling and field surveys to reappraise the geographic distribution and conservation status of the threatened thin-spined porcupine (Chaetomys subspinosus)
Source: PLoS One. 2018 Nov 27;13(11):e0207914. doi: 10.1371/journal.pone.0207914 (PMC6258534; doi:10.1371/journal.pone.0207914)
Supplement: S1 File — (PDF) [file pone.0207914.s003.pdf]

S1 Appendix. Differences between sympatric and allopatric porcupines from the Central Atlantic forest based on body weight, quills and external appearance.

Chaetomys subspinosus is a conspicuous species, easily discernible from sympatric and allopatric porcupine species *Coendou insidiosus* and *Coendou prehensilis*, respectively. These differences include variation in the size, quill flexibility and coloration, fur length and density, and nasofrontal sinus inflation [1,2]. Particularly, *C. subspinosus* and *C. insidiosus* are smaller and lighter (body weight~1.5 kg) [3] than *C. prehensilis* (body weight~4.5 kg) [4]. *C. subspinosus* differs from the sympatric small porcupine species *C. insidiosus* mainly by absence of the yellow-black-orange or yellow-black (tricolor or bicolor) defensive hard quills (Fig 1), characteristics of these and other porcupine species [1,5]. Different of the other porcupines, *Chaetomys subspinosus* have brown color and their pelage on the sides and rump is soft as bristles, doing not contributed to the physical defense of the animals [2,6]. The pelage on the head neck and anterior members is capable of piercing an aggressor, but don’t come loose when an aggressor attacks [2]. Finally, in contrast to *Coendou* species, the nasofrontal sinus of *Chaetomys* shows very little inflation.

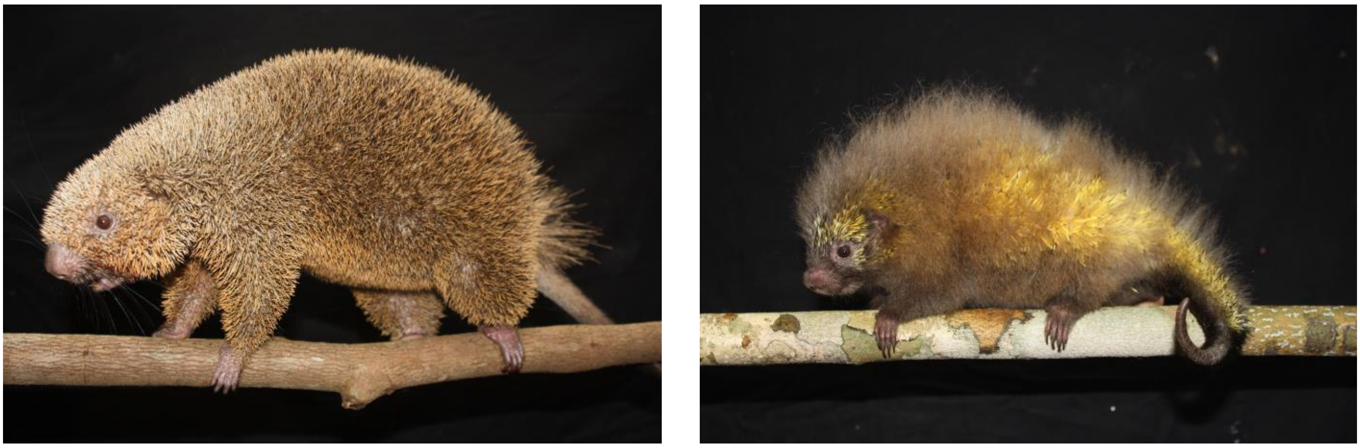

Fig 1. A view of the two sympatric porcupine species from the Central Atlantic forest: *Chaetomys subspinosus* (left) and *Coendou insidiosus* (right). (Photos: GAFG)

References

1. Voss RS, Hubbard C, Jansa SA. Phylogenetic Relationships of New World Porcupines (Rodentia, Erethizontidae): Implications for Taxonomy, Morphological Evolution, and Biogeography. Am Museum Novit. 2013;3769: 36pp.

2. Castilho LC, Martinez RA, Giné GAF, Ribeiro GC, Schiavetti A. The thin-spined porcupine, Chaetomys subspinosus (Rodentia : Erethizontidae), within protected areas in the Atlantic Forest , Brazil: local knowledge and threats. Trop Conserv Sci. 2013;6: 796–810.
3. Oliver WLR, Santos IL. Threatened endemic mammals of the Atlantic forest region of south-eastern Brazil. Jersey: Jersey Wildlife Preservation Trust Special Scientific 4; 1991.
4. Eisenberg JF, Redford KH. Mammals of the neotropics. Vol. 3. The Central Neotropics: Equador, Peru, Bolivia, Brazil. Chicago. Chicago.: The University of Chicago Press.; 1999.
5. Caldara Junior V, Leite YLR. Geographic variation in hairy dwarf porcupines of Coendou from eastern Brazil (Mammalia: Erethizontidae). Zool. 2012;29: 318–336. doi:10.1590/S1984-46702012000400005
6. Giné GAF, Duarte JMB, Motta TCS, Faria D. Activity, movement and secretive behavior of a threatened arboreal folivore, the thin-spined porcupine, in the Atlantic forest of southern Bahia, Brazil. Braae A, editor. J Zool. 2012;286: 131–139. doi:10.1111/j.1469-7998.2011.00855.x
