# Supplementary material for: Combining species distribution modeling and field surveys to reappraise the geographic distribution and conservation status of the threatened thin-spined porcupine (Chaetomys subspinosus)
Source: PLoS One. 2018 Nov 27;13(11):e0207914. doi: 10.1371/journal.pone.0207914 (PMC6258534; doi:10.1371/journal.pone.0207914)
Supplement: S1 Table — (DOCX) [file pone.0207914.s004.docx]

**S1 Table. Characterization of the remaining Atlantic forest in the potential extent of occurrence of the thin-spined porcupine (*Chaetomys subspinosus*) and zone of high climatic suitability predicted by modeling procedures.**

| Structural characteristics | Predicted species distribution | Adjusted species distribution | High-suitability zone |
| --- | --- | --- | --- |
| Total extent of occurrence (km^2^) | 112133 | 104327 | 16813 |
| Total remaining forested area (km^2^) | 14962 | 13870 | 3299 |
| Total number of forest fragments (n) | 24483 | 22476 | 4003 |
| Total protected forested area (km^2^) | 1310 | 1177 | 207 |
| % of forest cover in the extent of occurrence | 13.3 | 13.3 | 19.6 |
| % of protected forested area | 8.7 | 8.5 | 6.3 |
| % of ombrophilous forest area | 75.7 | 76.4 | 82.8 |
| % of seasonal forest area | 15.3 | 15.5 | 0.5 |
| % of *restinga* forest area | 9.0 | 8.1 | 16.7 |
| % of small forest fragments (< 50 ha) | 81.3 | 80.9 | 79.4 |
| % of medium forest fragments (50 to 250 ha) | 15.2 | 15.5 | 15.6 |
| % of medium-large forest fragments (250 to 1000 ha) | 2.9 | 3.0 | 3.7 |
| % of large forest fragments (> 1000 ha) | 0.6 | 0.6 | 1.2 |
